# PPAR*γ* Agonists: Potential as Therapeutics for Neovascular Retinopathies

**DOI:** 10.1155/2008/164273

**Published:** 2008-05-26

**Authors:** Harrihar A. Pershadsingh, David M. Moore

**Affiliations:** ^1^Department of Family Medicine, University of California, Irvine, CA 92797, USA; ^2^Department of Laboratory Medicine, University of California, San Francisco, CA 94143, USA; ^3^Department of Family Medicine, Kern Medical Center, Bakersfield, CA 93305, USA

## Abstract

The angiogenic, neovascular proliferative retinopathies, proliferative diabetic retinopathy (PDR), and age-dependent macular degeneration (AMD) complicated by choroidal neovascularization (CNV), also termed exudative or “wet” AMD, are common causes of blindness. The antidiabetic thiazolidinediones (TZDs), rosiglitazone, and troglitazone are PPAR*γ* agonists with demonstrable antiproliferative, and anti-inflammatory effects, in vivo, were shown to ameliorate PDR and CNV in rodent models, implying the potential efficacy of TZDs for treating proliferative retinopathies in humans. Activation of the angiotensin II type 1 receptor (AT1-R) propagates proinflammatory and proliferative pathogenic determinants underlying PDR and CNV. The antihypertensive dual AT1-R blocker (ARB), telmisartan, recently was shown to activate PPAR*γ* and improve glucose and lipid metabolism and to clinically improve PDR and CNV in rodent models. Therefore, the TZDs and telmisartan, clinically approved antidiabetic and antihypertensive drugs, respectively, may be efficacious for treating and attenuating PDR and CNV humans. Clinical trials are needed to test these possibilities.

## 1. INTRODUCTION

Angiogenesis and neovascularization involve formation and proliferation of new blood vessels and have a vital role normal growth and development, such as embryogenesis, wound healing, tissue repair [1, 2]. However, in pathological neovascularization, angiogenesis is aberrant and unregulated resulting in the formation of dysfunctional blood vessels [3]. The latter occurs in proliferative diabetic retinopathy (PDR) and choroidal neovascularization (CNV), “wet” or exudative age-dependent macular degeneration (AMD), wherein pathological neovascular vessels proliferate and leak fluid leading to retinal edema, subretinal and retinal/vitreous hemorrhage, retinal detachment, and blindness. In the United States, PDR is the most common preventable cause of blindness in adults <50 years [4], whereas CNV/AMD is the leading cause of blindness among people of European origin >65 years [5]. Both retinopathies are progressively destructive, leading to eventual and irreversible blindness. PDR is a serious microvascular complication of both type 1 and type 2 diabetes [6]. Type 2 diabetes is rapidly expanding worldwide and is estimated to reach 380 million by 2025 [7, 8]. PDR is progressive and compounded by persistent and substandard control of hyperglycemia, and concomitant cardiovascular risk factors, especially hypertension [9–11]. Nearly, all type 1 diabetics and >60% of type 2 diabetics have significant retinopathy after 20 years, emphasizing the need for more cost-effective therapy [6, 10, 11]. Hyperglycemia, advanced glycation end-products (AGEs), and hypoxia are believed to induce pathological angiogenesis and neovascularization within the retina [12]. Prevention of end-organ damage by early and aggressive diabetes management is the best approach to treating diabetic retinopathy (DR) [6, 12].

Visual acuity depends on a functional macula, located at the center of the retina where cone photoreceptors are most abundant. Exudative (wet) AMD is complicated by CNV, involving activation and migration of macrophages, and normally quiescent retinal pigment epithelial cells from the choroid and invasion of defective neovascular blood vessels into the subretinal space [13, 14]. Bleeding and lipid leakage from these immature vessels damage the retina and lead to severe vision loss and blindness [14, 15]. Current therapies of AMD are limited to treating the early stages of the disease, and include laser photocoagulation, photodynamic therapy, surgical macular translocation, and antiangiogenesis agents [13–16]. These invasive procedures are expensive, require repetition, whereas pharmacologic approaches could simplify therapy and reduce cost.

The peroxisome proliferator-activated receptor (PPAR) class of nuclear receptors (PPAR*α*, PPAR*β*/*δ*, and PPAR*γ*) belongs to the nuclear receptor superfamily that include the steroid, thyroid hormone, vitamin D, and retinoid receptors [17, 18]. In 1995, Lehmann et al. [19] discovered that PPAR*γ* was the intracellular high affinity receptor for the insulin-sensitizing, antidiabetic thiazolidinediones (TZDs), the activation of which also promotes growth arrest of preadipocytes, differentiation, adipogenesis, and differentiation into mature adipocytes [20]. Ligand activation of PPAR*γ* also downregulates the transcription of genes encoding inflammatory molecules, inflammatory cytokines, growth factors, proteolytic enzymes, adhesion molecules, chemotactic, and atherogenic factors [21–25] (Table 1).

Angiotensin II (AII) and components of the renin-angiotensin system (RAS) are expressed in the retina [26, 27]. AII promotes retinal leukostasis by activating the angiotensin type 1 receptor (AT1-R) pathway that propagates proinflammatory, proliferative mediators (Table 2) leading to the development and progression of PDR [28–30] and CNV [31]. By selectively blocking the AT1-R, angiotensin receptor blockers (ARBs) or “sartans,” for example, valsartan and telmisartan have been shown to confer neuroprotective and anti-inflammatory effects in animal models of retinal angiogenesis and neovascularization [32–36]. Among the seven approved ARBs, telmisartan and irbesartan were recently shown to constitute a unique subset of ARBs also capable of activating PPAR*γ* [37–39]. Valsartan and the remaining ARBs were inactive in the PPAR*γ* transactivation assay. In fact, telmisartan was shown to downregulate AT1 receptors through activation of PPAR*γ* [40]. Telmisartan was shown to provide therapeutic benefits in rodent models of PDR [33, 41–44] and CNV [45] but data with irbesartan is unavailable. Therefore, telmisartan and possibly irbesartan (data unavailable) may have enhanced efficacy in treating proliferative retinopathies. ARBs are safe and have beneficial cardiometabolic, anti-inflammatory, and antiproliferative effects. Among these telmisartan and irbesartan may have improved efficacy for targeting proliferative retinopathies. Table 3 provides relevant information on the various drugs described herein.

## 2. TISSUE DISTRIBUTION PPAR*γ*

Four PPAR*γ* mRNA isoforms have been identified [46] that encode two proteins, PPAR*γ*1 and PPAR*γ*2 [47, 48]. PPAR*γ*1 is the principal subtype expressed in diverse tissues, whereas PPAR*γ*2 predominates in adipose tissue [49, 50]. The PPAR*γ*2 protein differs from PPAR*γ*1 by the presence of 30 additional amino acids [49]. Tissue-specific distribution of isoforms and the variability of isoform ratios raise the possibility that isoform expression might be modulated by or reflect disease states in which PPAR*γ* activation or inactivation has a role. In humans, PPAR*γ* is most abundantly expressed mainly in white adipose tissue and large intestine, and to a significant degree in kidney, heart, small intestine, spleen, ovary, testis, liver, bone marrow, bladder, epithelial keratinocytes, and to a lesser extent in skeletal muscle, pancreas, and brain [51].

### 2.1. PPAR*γ* expression in the eye

PPAR*γ* is heterogeneously expressed in the mammalian eye [51–53]. PPAR*γ* was found to be most prominent in the retinal pigmented epithelium, photoreceptor outer segments, choriocapillaris, choroidal endothelial cells, corneal epithelium, and endothelium, and to a lesser extent, in the intraocular muscles, retinal photoreceptor inner segments and outer plexiform layer, and the iris [52]. Ligand-dependent activation of PPAR*γ* evokes potent inhibition of corneal angiogenesis and neovascularization [53–55]. The prominent expression of PPAR*γ* in selected tissues of the retina [52–54] provides the rationale for pharmacotherapeutic targeting of PPAR*γ* for treating ocular inflammation and proliferative retinopathies [53–56].

### 2.2. Importance of PPAR*γ* in proliferative retinopathy

To determine whether endogenous PPAR*γ* played a role in experimental DR, Muranaka et al. [54] evaluated retinal leukostasis and retinal (vascular) leakage in streptozotocin-induced diabetic C57BL/6 mice deficient in PPAR*γ* expression (heterozygous genotype, PPAR*γ*+/−) after 120 days. Retinal leukostasis and leakage were greater (205% and 191%, resp.) in the diabetic PPAR*γ*+/− mice, compared to diabetic wild-type (PPAR*γ*+/+) mice. In streptozotocin-induced diabetic Brown Norway rats, oral administration of the TZD PPAR*γ* ligand, rosiglitazone for 21 days (3 mg/kg body weight/day, initiated post-streptozotocin injection) resulted in suppression of retinal leukostasis by 60.9% (*P* < .05), and retinal leakage by 60.8% (*P* < .05) [54]. Expression of the inflammatory molecule. ICAM-1 protein was upregulated in the retina of the rosiglitazone-treated group, though the levels of VEGF and TNF-*α* were unaffected [54]. These findings provide strong evidence for a role of PPAR*γ* activity in the pathogenesis of DR and provide novel genomic information that therapeutic targeting of PPAR*γ* with a known PPAR*γ* ligand, the TZD rosiglitazone, can attenuate the progression of PDR. Whether a similar effect may apply to the prevention or attenuation of CNV is currently unknown and should be explored.

## 3. ANTIDIABETIC THIAZOLIDINEDIONES (TZDs) AND PROLIFERATIVE RETINOPATHIES

The insulin-sensitizing TZDs, rosiglitazone, and pioglitazone are approved for the treatment of type 2 diabetes. Because they increase target tissue sensitivity to insulin without increasing insulin secretion [57], there is no risk of hypoglycemia, though there is a risk fluid retention in diabetic patients, especially those with coexisting heart failure, or at risk for developing CHF [58].

By activating PPAR*γ*, TZDs modulate groups of genes involved in energy metabolism [59], inflammation, and cellular differentiation [60–64] by down-regulating the activity of the proinflammatory nuclear receptors (NF-*κ*B, AP-1, STAT, NFAT), and inhibiting the activity and expression of inflammatory cytokines (TNF-*α*, IL-1*β*, IL-2, IL-6), iNOS, proteolytic enzymes (MMP-3 and MMP-9), and growth factors (VEGF, PDGF-BB, bFGF, EGF, TGF-*β*) (Table 1). Because of these broadly beneficial and protective actions of PPAR*γ* agonists, TZDs have been under development for the treatment of conditions beyond type 2 diabetes, including atherosclerosis [64, 65], psoriasis [66], inflammatory colitis [67], nonalcoholic steatohepatitis [68], and Alzheimer’s disease [69]. More recently, TZDs have been found to protect against glutamate cytotoxicity in retinal ganglia and have antioxidant properties [70] suggesting that PPAR*γ* agonists could prove valuable in targeting retinal complications [71].

### 3.1. Therapeutic effects on proliferative diabetic retinopathy (PDR)

Retinal capillaries consist of endothelial cells, basement membrane neovascularization, and intramural pericytes within the basement membrane which are important in vascular development and maturation [44]. Selective loss of pericytes from the retinal capillaries characteristically occurs early in diabetic retinopathy (DR) [72]. Diabetic macular edema (DME), often associated with PDR, involves breakdown of the blood-retinal barrier and leakage of plasma from blood vessels in the macula causing macular edema and impaired vision [73, 74]. Resorption of the fluid from plasma leads to lipid and lipoprotein deposition forming hard exudates [75]. In PDR, inflammation leads to endothelial dysfunction, retinal vascular permeability, vascular leakage, and adhesion of leukocytes to the retinal vasculature (leukostasis), progressive capillary nonperfusion, and DME [12]. Intraretinal microvascular abnormalities and progressive retinal ischemia lead to neovascular proliferation within the retina, bleeding, vitreous hemorrhage, fibrosis, and retinal detachment [74–76]. Despite advancements in ophthalmologic care and the management of both type 1 and type 2 diabetes, PDR remains a leading cause of preventable blindness [5–7]. Primary interventions, especially intensive glycemic and blood pressure control, and management of other cardiovascular risk factors are essential [6, 73–75]. Focal laser photocoagulation remains the only surgical option for reducing significant visual loss in eyes with macular edema [6, 9–12]. The risk of blindness with untreated PDR is currently greater than 50% at 5 years, but can be reduced to less than 5% with appropriate therapy [5–7]. At present, there is insufficient evidence for the efficacy or safety of pharmacological interventions, including therapy targeting vascular endothelial growth factor (i.e., anti-VEGF antibody therapy), though intravitreal glucocorticoids may be considered when conventional treatments have failed [6, 12].

Troglitazone and rosiglitazone were shown to attenuate VEGF-induced retinal endothelial cell proliferation, migration, tube formation, and signaling, in vitro [55] by arresting the growth cycle of endothelial cells [62]. Local intrastromal implantation of micropellets containing pioglitazone into rat corneas significantly decreased the density of VEGF-induced angiogenesis, an accepted animal model of retinal neovascularization [53].

Adverse conditions that contribute to macular edema and retinal degeneration in PDR include generation of advanced glycation end products (AGEs), local ischemia, oxidative reactions, and hyperglycemia-induced toxicity [72, 75, 76]. In PPAR*γ*-expressing retinal endothelial cells, troglitazone, and rosiglitazone inhibited VEGF-stimulated proliferation, migration, and tube formation [55, 77]. The effects of troglitazone and rosiglitazone were also evaluated in the oxygen-induced ischemia murine model of retinal neovascularization, an experimental model of PDR [77]. Although the model lacks specific metabolic abnormalities found in diabetes, it isolates the VEGF-driven process in which neovascularization is stimulated by increased VEGF expression in the inner retina [77]. Both troglitazone and rosiglitazone decreased the number of microvascular tufts induced on the retinal surface, suggesting inhibition of an early aspect of neovascularization. The inhibitory effects were dose-dependent (IC_50_ ≃ 5 *μ*mo1/L) [77]. These findings support the proposal that TZDs may have beneficial effects by reducing or delaying the onset of PDR in diabetic patients. Prospective clinical trials are required to demonstrate clinical efficacy.

### 3.2. Therapeutic effects on choroidal neovascularization (CNV)

AMD complicated with CNV involves angiogenesis and neovascularization in the choroid with hemorrhage in the subretinal space, fluid accumulation beneath the photoreceptors within the fovea, and neural cell death in the outer retina [13–16]. CNV is present with vascular inflammation, unbridled vascular proliferation, aberrant epithelial and endothelial cell migration, and inappropriate production of proinflammatory cytokines, inducible nitric oxide synthase, growth factors, proteolytic enzymes, adhesion molecules, chemotactic factors, atherogenic, and other mediators that propagate defective blood vessel proliferation [5, 13–16, 78]. Elevated blood pressure, serum lipids, smoking, and insulin resistance also have an etiological role in CNV development [78]. Therefore, control of cardiometabolic risk factors is important in palliative management of CNV [79, 80]. Recently, therapy for early exudative AMD has been directed toward intravitreal injection of VEGF-directed antibodies or fragments thereof [14–16]. However, excessive cost ($1,950/dose) is a major issue [http://www.globalinsight.com/SDA/SDADetail6273.htm]. Monthly treatments are difficult for patients to tolerate, and the risk of serious adverse effects increases over time [16]. On the other hand, synthetic, nonpeptide PPAR*γ* agonists [81, 82] are straightforward to synthesize, inexpensive to formulate.

CNV comprises the underlying pathology of exudative AMD, principally involving the subretinal vasculature and choriocapillaris, leading to capillary closure and retinal ischemia, angiogenesis, retinal neovascularization, bleeding into the vitreous, retinal detachment and degeneration, and eventually vision loss [13–16]. PPAR*γ* is expressed in the choriocapillaris, choroidal endothelial cells, retinal endothelial cells, and retinal pigmented epithelium [52, 83]. VEGF is a potent inducer of retinal [13–16] angiogenesis and neovascularization. In their landmark study, Murata et al. [83] demonstrated the expression of PPAR*γ*1 in human retinal pigment epithelial (RPE) cells and bovine choroidal endothelial cells (CECs), and that application of the TZDs troglitazone or rosiglitazone (0.1–20 *μ*mol/L) inhibited VEGF-induced proliferation and migration of RPE and CEC cells, and neovascularization [83]. Moreover, in the eyes of rat and cynomolgus monkeys in which CNV was induced by laser photocoagulation, intravitreal injection of troglitazone markedly inhibited CNV compared to control eyes (*P* < .001). The treated lesions showed significantly less fluorescein leakage and were histologically thinner in troglitazone-treated animals, without adverse effects in the adjacent retina or in control eyes [83]. These findings suggest that pharmacological activation of PPAR*γ* by TZDs appear to have a palliative or therapeutic effect on experimental CNV. Again, clinical trials are required to demonstrate efficacy in the clinical setting.

### 3.3. Adverse effects of TZDs: fluid retention and macular edema

Pioglitazone and rosiglitazone are generally safe though, in type 2 diabetic patients, there is a risk of weight gain (1–3 kg) and fluid retention [58]. The incidence of peripheral edema is greater in those concurrently taking exogenous insulin, increasing from 3.0–7.5% to 14.7–15.3% [58]. The edema may be related to TZD-induced vasodilation, increased plasma volume secondary to renal sodium reabsorption, and reflex sympathetic activation [58]. The association of rosiglitazone treatment with development of macular edema has been reported [84]. In a case review of 11 patients who developed peripheral and macular edema, while on the TZD therapy [85] 8 patients experienced resolution of macular edema with improved vision, without laser treatment, 3 months to 2 years after TZD cessation. Therefore, DME should be considered in type 2 diabetic patients treated with a TZD, especially those with peripheral edema, or other symptoms or risk factors of CHF, or concurrently taking exogenous insulin or nitrates. Drug cessation usually results in rapid resolution of both peripheral and macular edema [85].

## 4. ANTIHYPERTENSIVE ANGIOTENSIN RECEPTOR BLOCKERS (ARBs) THAT ACTIVATE PPAR*γ*

In their search for PPAR*γ* agonists that lack the adverse effects of TZDs, Benson et al. [37] screened the active forms of all currently available antihypertensive “sartans” (ARBs): losartan, valsartan eprosartan, irbesartan, candesartan, telmisartan, and olmesartan, using the standard GAL-4 cell-based PPAR*γ* transactivation assay. Only telmisartan and irbesartan [37, 38] activated PPAR*γ* and promoted adipogenesis, intracellular lipid accumulation and differentiation of preadipocyte fibroblasts into mature adipocytes, in vitro, hallmark properties of PPAR*γ* agonists [19]. The EC_50_ values for transactivation of PPAR*γ* by telmisartan and irbesartan were 4.5 *μ*mol/L and 27 *μ*mol/L, respectively [37–39] (Table 3). Although the PPAR*γ* transactivation assay may not recapitulate conditions in vivo, based on pharmacokinetic considerations, concentrations of these ARBs required to activate PPAR*γ* in vivo are achievable by standard dosing [86, 87]. By functioning as partial PPAR*γ* agonists this unique subset of ARBs may provide added end-organ benefits in certain patient populations such patients with the metabolic syndrome [87] and other cardiometabolic risk factors, including atherosclerosis, atherogenesis, and may have palliative effects on proliferative retinopathies.

ARBs bear an acidic group (tetrazole or carboxyl group) at the *ortho* position on the terminal benzene ring of the biphenyl moiety, which is essential for AT1 receptor binding. Telmisartan bears a carboxyl and irbesartan, a tetrazole [87, 88]. The active forms of all other ARBs have two acidic groups at opposite molecular poles. This second acidic group limits accessibility, and hinders binding to the hydrophobic region of the PPAR*γ* receptor [87, 88]. Therefore, among currently available ARBs, the molecular dipole appears to be an important structure-functional determinant of ligand binding to the PPAR*γ* receptor [87]. Compared to all other ARBs, telmisartan has a uniquely long elimination half-life (24 hours), and the largest volume of distribution (500 L, and >10-fold in excess of other ARBs) which greatly increases central bioavailability upon oral dosing [86]. Furthermore, telmisartan has been shown to have significant anti-inflammatory and antioxidant activity, which may enhance its effectiveness in attenuating the progression of proliferative retinopathies [89–91].

### 4.1. Full versus partial PPAR*γ* agonists

The PPAR*γ* receptor is composed of five different domains, an N-terminal region or domain A/B, a DNA binding domain C (DBD), a hinge region (domain D), a ligand binding domain E (LBD), and a domain F [81, 92, 93]. The A/B domain contains an activation function-1 (AF-1) that operates in absence of ligand. The DBD confers DNA binding specificity. PPAR*γ* controls gene expression by binding to specific DNA sequences or peroxisome proliferation-responsive elements (PPREs) in the regulatory region of PPAR-responsive genes. The large LBD (∼1300 Å^3^) allows the receptor to interact with a broad range of structurally distinct natural and synthetic ligands [81, 92, 93]. The receptor protein contains 13 helices, and the activation function, AF-2 helix located in the C terminus of the LBD is intimately integrated with the receptor's coactivator binding domain [81]. Ligand-dependent stabilization is required for activation of the downstream transcriptional machinery [81, 92, 93].

Thiazolidinedione full agonists (TZDfa), for example, rosiglitazone and pioglitazone permit certain coactivators to interact with the PPAR-LBD in an agonist-dependent manner and are oriented by a “charge clamp” formed by residues within helix 3 and the AF-2 arm of helix 12 in the LBD [45, 93]. Based on protease digest patterns and crystallographic findings, the PPAR*γ* non-TZD partial agonist (nTZDpa) [94] and PPAR*γ* partial agonist/antagonist, GW0072 [95] are mainly stabilized by hydrophobic interactions with helixes H3 and H7.

**Figure 1:**
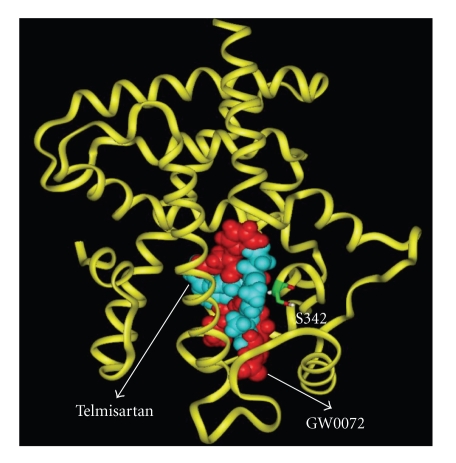
Telmisartan (blue) superimposed on the co-crystal structure of GW0072 (red) bound within the PPAR*γ*-LBD. Telmisartan and GW0072 are Van der Waals space-filling representations, and the protein backbone by the yellow ribbon. Formation of hydrogen bonds and interactions between both ligands and the amide proton of Ser342 contribute toward stabilization of the partial agonists within the PPAR*γ*-LBD. (*Kindly provided by Dr. P.V. Desai & Professor M.A. Avery, Department of Medicinal Chemistry, University of Mississippi, USA*.)

**Figure 2:**
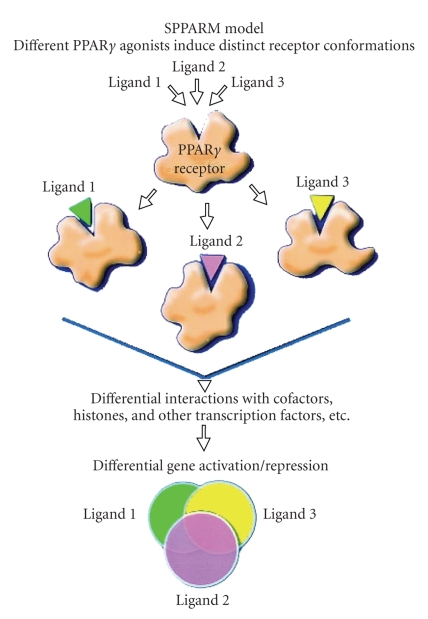
Selective PPAR*γ* modulator (SPPAR*γ*M) model of PPAR*γ* ligand action. PPAR*γ* is a multivalent receptor whose ligand binding domain can accommodate different PPAR*γ* ligands. Ligands 1, 2, or 3 (e.g., full agonist, partial agonist, or SPPAR*γ*M) are capable of inducing distinct receptor combinations leading to selective gene expression. Each ligand-receptor complex assumes a somewhat different three-dimensional conformation, leading to unique and differential interactions with cofactors, histones (acetylases/deacetylases), and other transcription factors. Consequently, each PPAR*γ* ligand-receptor complex leads to a differential, but overlapping, pattern of gene expression. Thus, each ligand will activate, or repress multiple genes leading to differential overlapping expression of different sets of genes. (Adapted with permission from: J. M. Olefsky, “Treatment of insulin resistance with peroxisome proliferator-activated receptor gamma agonists.” Journal of Clinical Investigation, vol. 106, no. 4, pp. 467-472, 2000); H. A. Pershadsingh, “Treating the metabolic syndrome using angiotensin receptor antagonists that selectively modulate peroxisome proliferator-activated receptor-gamma.” International Journal of Biochemistry and Cellular Biology, vol. 38, nos 5-6, pp. 766-781, 2006.)

The antihypertensive ARBs telmisartan and irbesartan have been shown to function as partial PPAR*γ* agonists, similar to the previously identified nTZDpa [94]. Based on molecular motifs, telmisartan appears to occupy a region in proximity with helix 3, with key interactions between the carboxylic acid group of the ligand and Ser342 near the entrance of the PPAR*γ* pocket [37] (Figure 1). Telmisartan and irbesartan appear to cause an alteration in the conformation of these helixes similar to that induced by nTZDpa [37, 39], promoting differences in receptor activation and target gene expression that confer a low adipogenic potential compared with full agonists (TZDfa) like rosiglitazone and pioglitazone, which are known to have a high adipogenic potential and promote weight gain [58, 81, 94]. Differential binding motifs reflecting full versus partial PPAR*γ* agonism are illustrated in Figure 2.

**Figure 3:**
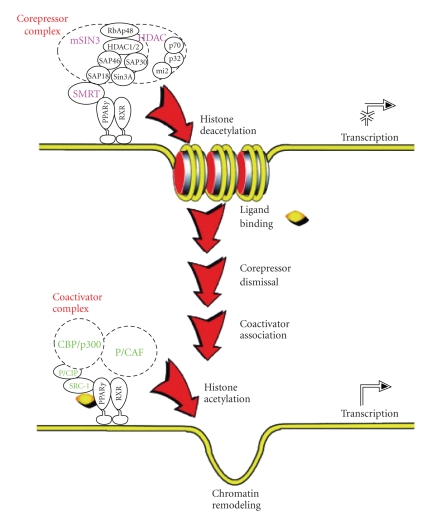
Schematic diagram of the mechanisms of PPAR*γ* action. In the unliganded state (top), the PPAR*γ* receptor exists as a heterodimer with the RXR nuclear receptor and the heterodimer is located on a PPAR response element (PPRE) of a target gene. The unliganded receptor heterodimer complex is associated with a multicomponent corepressor complex, which physically interacts with the PPAR*γ* receptor through silencing mediator for retinoid and thyroid hormone receptors (SMRT). The corepressor complex contains histone deacetylase (HDAC) activity, and the deacetylated state of histone inhibits transcription. After PPAR*γ* ligand binding, the corepressor complex is dismissed, and a coactivator complex is recruited to the heterodimer PPAR*γ* receptor (bottom). The coactivator complex contains histone acetylase activity, leading to chromatin remodeling, facilitating active transcription. (Adapted with permission from: J. M. Olefsky, “Treatment of insulin resistance with peroxisome proliferator-activated receptor gamma agonists.” Journal of Clinical Investigation, vol. 106, no. 4, pp. 467-472, 2000); C. K. Glass, M. G. Rosenfeld, “The coregulator exchange in transcriptional functions of nuclear receptors”. Genes & Development,vol. 14, no. 2, pp. 121-141, 2000.)

Several coactivators, including CREB-binding protein complex, CBP/p300, steroid receptor coactivator (SRC)-1, nuclear receptor corepressor (NcoR), DRIP204, PPAR binding protein (PBP)/TRAP220, and PPAR*γ* coactivator-1 (PGC-1), among others, interface functionally between the nuclear receptor and the transcription initiation machinery in ways not well understood [94]. Differential ligand-induced initiation of transcription is the consequence of differential recruitment and release of selective coactivators and corepressors [96] (Figure 3). For example, NcoR a silencing mediator when bound to PPAR*γ* suppresses adipogenesis in the absence of ligand. Activation by TZDfa ligands causes release of NcoR and recruitment of the nuclear receptor coactivator complex, NcoA/SRC-1 which promotes adipogenesis and lipid storage [94].

Demonstration of direct interaction between telmisartan or irbesartan with PPAR*γ* protein, by analyzing migration patterns of ligand-PPAR*γ* protein fragments in trypsin digestion experiments, indicated that both ARBs downregulated PPAR*γ* mRNA and protein expression in 3T3-L1 human adipocytes, a known property of PPAR*γ* ligands in adipocytes [39]. In fact, both telmisartan and irbesartan caused release of NCoR and recruitment of NCoA/DRIP205 to PPAR*γ* in a concentration-dependent manner [39]. The transcription intermediary factor 2 (TIF-2), an adipogenic coactivator implicated in PPAR*γ*-mediated lipid uptake and storage, which increased the transcriptional activity of PPAR*γ*, was potentiated by pioglitazone but not by the ARBs [39]. Moreover, irbesartan and telmisartan also induced PPAR*γ* activity in an AT1R-deficient cell model (PC12W), demonstrating that their effects on PPAR*γ* activity were independent of their AT1-R blocking actions [38]. These data demonstrate the functional relevance of selective cofactor docking by the ARBs, and compared to pioglitazone, identify telmisartan and irbesartan as unique selective PPAR*γ* modulators (SPPAR*γ*Ms) that can retain the metabolic efficacy of PPAR*γ* activation, while reducing adverse effects, in parallel AT1-R blockade [37–39, 88]. Therefore, as dual ARB/SPPAR*γ*M ligands, telmisartan and irbesartan have important differential effects on PPAR*γ*-dependent regulation of gene transcription, without the limitations of fluid retention and weight gain, providing improved therapeutic efficacy by combining potent antihypertensive, antidysmetabolic, anti-inflammatory, and antiproliferative actions in the treatment of the proliferative retinopathies.

### 4.2. Expression of the renin-angiotensin system in the eye

The RAS evolved to maintain volume homeostasis and blood pressure through vasoconstriction, sympathetic activation, and salt and water retention [97]. AII binds and activates two primary receptors, AT1-R, and AT2-R. In adult humans, activation of the AT1-R dominates in pathological states, leading to hypertension, atherosclerosis, cardiac failure, end-organ demise (e.g., nephropathy), and proliferative retinopathies. AT2-R activation generally has beneficial effects, counterbalancing the actions propagated through AT1-R. ARBs selectively block AT1-R, leaving AII to interact with the relatively beneficial AT2-R. AII is generated in cardiovascular, adipose, kidney, adrenal tissue, and the retina; and through AT1-R activation promotes cell proliferation, migration, inflammation, atherogenesis, and extracellular matrix formation [97].

AII and genes enconding angiotensinogen, renin, and angiotensin converting enzyme (ACE) have been identified in the human neural retina [98]. Prorenin and renin have been identified in diabetic and nondiabetic vitreous, and intravitreal prorenin is increased in PDR [99]. Angiotensin I and AII were found to be present in ocular fluids of diabetic and nondiabetic patients [100]. AII and VEGF have been identified in the vitreous fluid of patients with PDR [101], and AT1 and AT2 were identified in the neural retina [102]. Furthermore, AT1 and AT2, AII, and its bioactive metabolite Ang-(1–7) were identified in blood vessels, pericytes, and neural (Müller) cells suggesting that these glial cells are able to produce and process AII [102]. Thus, AII signaling via the AT1 pathway within the retina may mediate autoregulation of neurovascular activity, and the onset and severity of retino-vascular disease [103].

### 4.3. Pathophysiological role of AT1 activation in proliferative retinopathies

AT1 activation participates in the pathogenesis of PDR, involving inflammation, oxidative stress, cell hypertrophy and proliferation, angiogenesis, and fibrosis [101, 103]. The RAS is upregulated concomitant with hypoxia-induced retinal angiogenesis [102–104] and is linked to AII-mediated induction of inflammatory mediators and growth factors, including VEGF and PDGF [103–106]. AT1 blockade with candesartan inhibited pathological retinopathy in spontaneously diabetic Torii rats by reducing the accumulation of the advanced glycation end-product (AGE) pentosidine [34]. AGEs contribute to vascular dysfunction by increasing the activity of VEGF and reactive oxygen species [34]. Treatment with candesartan reduced the accumulation pentosidine and VEGF gene expression in the diabetic rat retina [34]. AT1-R, AT2-R, and AII were shown to be expressed in the vascular endothelium of surgical samples from human CNV tissues and chorioretinal tissues from mice in which CNV was laser-induced [40]. Therefore, the retinal RAS appears to have an important pathophysiological role in proliferative retinopathies.

### 4.4. Therapeutic effects of telmisartan on PDR and CNV

AII is among the most potent vasopressive hormones known and contributes to the development of leukostasis in early diabetes [29]. Hypertension increases retinal inflammation and exacerbates oxidative stress in experimental DR [34, 107], and in diabetic hypertensive rats, prevention of hypertension abrogaItes retinal inflammation and leukostasis in early DR [108]. Therefore, RAS blockade by the dual ARB/PPAR*γ* agonists, telmisartan or irbesartan, may have enhanced effects for abrogating inflammatory and other pathological events that contribute to or exacerbate PDR and CNV/AMD. In clinical studies, reduction of hypertension by any means reduces the risk of development and the progression of DR [109]. ARBs are widely used antihypertensive agents clinically.

Induction of diabetes by streptozotocin injection in C57BL/6 mice caused significant leukostasis and increased retinal expression and production of AII, AT1-R, and AT2-R [30]. Intraperitoneal administration of telmisartan inhibited diabetes and glucose-induced retinal expression of ICAM-1 and VEGF, and upregulation of ICAM-1 and MCP-1, via inhibition of nuclear translocation of NF-*κ*B [33]. There have been no reports on the effects of irbesartan on PDR or CNV/AMD.

In the laser-induced mouse model of CNV, new vessels from the choroid invade the subretinal space after photocoagulation, reflecting the choroidal inflammation and neovascularization seen in human exudative AMD. Based a recent suggestion [110], Nagai et al. [45] evaluated and compared the effects of telmisartan with valsartan, an ARB lacking significant PPAR*γ* activity [38, 39], and suitable control to evaluate the role of telmisartan PPAR*γ* activity. Both ARBs have identical affinities for the AT1-R (∼10 nmo1/L) [97]. Telmisartan (5 mg/kg, i.p.) or valsartan (10 mg/kg, i.p.) significantly suppressed CNV in mice [45]. Simultaneous administration of the selective PPAR*γ* antagonist GW9662, partially (22%) but significantly reversed the suppression of CNV in the group receiving telmisartan but not the group receiving valsartan [45], indicating separate beneficial contributions via AT1 blockade and PPAR*γ* activation, respectively [45]. Using GW9662, similar findings were obtained identifying participation of PPAR*γ* in the suppressive effect of telmisartan on the inflammatory mediators, ICAM-1, MCP-1, VEGFR-1 in b-End3 vascular endothelial cells, and VEGF and in RAW264.7 macrophages, unrelated to AT1 blockade [45]. These findings confirm that the beneficial effects of telmisartan are derived from a combination of AT1 blockade and PPAR*γ* activation. The inhibitory effects of valsartan were insensitive to the presence of GW9662. This is the first known demonstration of PPAR*γ*-dependent inhibitory actions of a non-TZD PPAR*γ* agonist on CNV. There have been no reports on the effects of irbesartan on PDR or CNV.

### 4.5. Therapeutic potential of dual ARB/SPPAR*γ*Ms

Reduction in the cardiometabolic risk profile by lowering high blood pressure, improving insulin sensitivity, normalizing the lipid profile, and inhibiting inflammatory pathways are known to impede the pathological evolution of proliferative retinopathies. The dual ARB/SPPAR*γ*M ligands, telmisartan has been shown to be effective in this regard in the rodent model, though irbesartan has yet to be tested experimentally. PPAR*γ* activation has beneficial effects by lowering hyperglycemia and improving the metabolic profile in individuals with type 2 diabetes and the metabolic syndrome. The fact that both AT1-R blockade and PPAR*γ* activation by telmisartan had independent synergistic effects in the murine model of laser-induced CNV is an important finding [40]. It would be useful to test whether irbesartan has effects similar to those of telmisartan in animal models of PDR and CNV/AMD [28, 31–34, 40], as both ARBs similarly attenuate inflammation, proliferation, and improve the metabolic syndrome [111, 112]. Also, unlike TZDs, telmisartan (but not valsartan) increases caloric expenditure and protects against weight gain and hepatic steatosis [113]. With its high lipid solubility, large volume of distribution, and other favorable pharmacokinetic properties [86–88], telmisartan may be effective when administered orally. If oral delivery proves therapeutically ineffective, the drug may be formulated for administration via implant or transscleral application for local delivery to the posterior segment [114–116].

## 5. CONCLUDING REMARKS

Hypertension, insulin resistance, dyslipidemia, and risk for atherosclerosis and atherogenesis, all components of the metabolic syndrome, comprise significant epidemiologic risk factors for neovascular, proliferative retinopathies [6, 9, 12, 117, 118]. Photodynamic and anti-VEGF therapy, current treatments for CNV/AMD are cost-intensive. Treatments for PDR are limited to surgical options in advanced disease when the visual function is irreversibly affected [3–6, 14–16]. Therefore, alternative, low cost, prophylactic and/or palliative pharmacotherapeutic approaches are attractive and desirable. The currently approved antidiabetic TZD, rosiglitazone (a full PPAR*γ* agonist), and the antihypertensive ARB, telmisartan (a partial PPAR*γ* agonist) have both shown promise in animal models of proliferative retinopathies. The potential efficacies of irbesartan in proliferative retinopathies remain to be determined. Administration of TZDs may, in patients with AMD, slow the progression to CNV, and in patients with diabetic retinopathy attenuate the progress to PDR, provided that: (1) their risk of macular edema is low, (2) they lack symptoms of CHF or cardiomyopathy, and (3) are not taking insulin or nitrates. The efficacy and safety limitations of the TZDs are well understood [119–123] and their use would require careful benefit-to-risk analysis. Because these drugs have been in use clinically for a decade, well-designed retrospective analyses in carefully selected patient populations may reveal useful information regarding their clinical potential.

Several SPPAR*γ*Ms currently which are under development for treating type 2 diabetes [124] could be screened in animal models of PDR and CNV to determine their potential efficacy for treating proliferative retinopathies. Long-term, prospective clinical trials are needed to demonstrate the efficacy of currently approved TZDs and ARBs (Table 3). Notably, three large prospective phase III trials are underway to evaluate the effect of the ARB, candesartan on retinopathy in normotensive type 1 and type 2 diabetes patients, the diabetic REtinopathy candesartan trials (DIRECTs) Programme [125]; estimated study completion date: June 2008. These studies will provide important insight into the potential efficacy of ARBs in general in the treatment of DR. With their capacity for activating PPAR*γ* and improving the metabolic profile, the clinical efficacy of telmisartan and possibly irbesartan could be evaluated in patients at risk for developing PDR and CNV, especially those with deficiencies in carbohydrate and lipid metabolism. Moreover, with their unique structure/activity profile, these compounds may provide a drug discovery platform for designing therapeutic agents for treating proliferative retinopathies.

## Figures and Tables

**Table 1:** Growth factors, cytokines, chemokines, and other proinflammatory mediators downregulated by PPAR*γ* activation. PDGF-BB, platelet-derived growth factor-BB homodimer; AP-1, activated protein-1; NF-*κ*B = nuclear factor-*κ*B; NFAT = nuclear factor of activated T lymphocytes; STAT = signal transducer and activator of transcription; ICAM, intracellular adhesion molecule; VCAM, vascular cell adhesion molecule; iNOS, inducible nitric oxide synthase. (Adapted with permission from: B. Staels, “PPAR*γ* and atherosclerosis.” Current Medical Research and Opinion, vol. 21, Suppl. 1, pp. S13-S20, 2005; H. A. Pershadsingh, “Dual peroxisome proliferator-activated receptor-alpha/gamma agonists : in the treatment of type 2 diabetes mellitus and the metabolic syndrome.” Treatments in Endocrinology, vol. 5, no. 2, pp. 89-99, 2006.)

Growth factors	Cytokines	Chemokines	Nuclear transcription factors	Other molecules
ATII	IL-1*β*	IL-8	AP-1	IFN-*γ*
TGF-*β*	IL-2	MCP-1	NF-*κ*B	iNOS
ET-1	IL-6	RANTES	STAT	PAI-1
bFGF	TNF-*α*		NFAT	MMP-2
PDGF-BB				MMP-9
EGF				VCAM-1
VEGF				ICAM-1
				E-selectin

**Table 2:** Growth factors, cytokines, chemokines, and other proinflammatory mediators upregulated by angiotensin II stimulation. ET-1, endothelin-1; TGF-*β*, transforming growth factor-*β*; CTGF, connective tissue growth factor; bFGF, basic fibroblast growth factor; PDGF-AA, platelet-derived growth factor-AA homodimer; EGF, epidermal growth factor; VEGF, vascular endothelial cell growth factor; IL, interleukin; GM-CSF, granulocyte-macrophage colony-stimulating factor; TNF-*α*, tumor necrosis factor-*α*; MCP-1, monocyte chemoattractant protein-1; MIP, macrophage inflammatory protein; NF-*κ*B, nuclear factor-*κ*B; NFAT, nuclear factor of activated T lymphocytes; STAT, signal transducer and activator of transcription; RANTES, regulated on activation, normal T-cell expressed and secreted; IFN-*γ*, interferon-*γ*; PAI-1, plasminogen activator inhibitor type 1; AP-1, activated protein-1. (Adapted with permission from: R. E. Schmieder, K. F. Hilgers, M. P. Schlaich, B. M. Schmidt, “Renin-angiotensin system and cardiovascular risk.” Lancet, vol. 369, no. 9568, pp. 1208-1219, 2007.)

Growth factors	Cytokines	Chemokines	Other proinflammatory molecules
ET-1	IL-1*β*	IL-8	IFN-*γ*
TGF-*β*	IL-6	MCP-1	Tissue factor
CTGF	IL-18	MIP-1	PAI-1
bFGF	GM-CSF	RANTES	
PDGF-AA	TNF-*α*		
EGF			
VEGF			

**Table 3:** Comparison of pharmacological and other relevant properties of thiazolidinedione (TZD) full PPAR*γ* agonists and dual angiotensin II type 1 receptor blocker/selective PPAR*γ* modulator (ARB/SPPAR*γ*M).

Parameter	TZDs^†^			ARBs*	
	Troglitazone	Pioglitazone	Rosiglitazone	Telmisartan	Irbesartan
Primary pharmacological target	PPAR*γ*	PPAR*γ*	PPAR*γ*	AT1-R	AT1-R
Type of PPAR*γ* agonists	Full PPAR*γ* agonists			Selective PPAR*γ* modulator (SPPAR*γ*M)	
Drug class (common names)	Thiazolidinedione (TZDs)			Angiotensin receptor blockers (ARBs)	
PPAR*γ* activation (EC_50_ in *μ*M)	0.55	0.58	0.043	4.5	27
Therapeutic indication	Treatment of type 2 diabetes mellitus			Treatment of hypertension	
Primary therapeutic mechanism	Increase insulin sensitivity			Lower blood pressure	
Serious adverse effect (Black box warning)	Fluid retention/weight gain/heart failure			None	None
Supplier/Pharmaceutical Co.	Sigma-Aldrich, St. Louis, Mo, USA	Takeda Pharmaceuticals North America, Deerfield, Ill, USA	GlaxoSmithKline, NC, USA	Boehringer- Ingelheim Pharmaceuticals, Inc., Ridgefield, Conn, USA	Sanofi-Aventis, Bridgewater, NJ, USA

^†^Thiazolidinedione full PPAR*γ* agonists; troglitazone was withdrawn from the market (1998) because of association with rare cases of fatal hepatic failure. Rosiglitazone and pioglitazone have no such known association. *Other FDA-approved ARBs had EC_50_ values > 100*μ*M (see [37, 38]). EC_50_ values shown were determined using the standard PPAR*γ*-GAL4 transactivation assays.
